# Relative Movements of Domains in Large Molecules of the Immune System

**DOI:** 10.1155/2015/210675

**Published:** 2015-12-21

**Authors:** Wolfgang Schreiner, Rudolf Karch, Reiner Ribarics, Michael Cibena, Nevena Ilieva

**Affiliations:** ^1^Section of Biosimulation and Bioinformatics, Center for Medical Statistics, Informatics and Intelligent Systems (CeMSIIS), Medical University of Vienna, Spitalgasse 23, 1090 Vienna, Austria; ^2^Institute of Information and Communication Technologies (IICT), Bulgarian Academy of Sciences, 25 A, Acad. G. Bonchev Street, 1113 Sofia, Bulgaria

## Abstract

Molecular dynamics was used to simulate large molecules of the immune system (major histocompatibility complex class I, presented epitope, T-cell receptor, and a CD8 coreceptor.) To characterize the relative orientation and movements of domains local coordinate systems (based on principal component analysis) were generated and directional cosines and Euler angles were computed. As a most interesting result, we found that the presence of the coreceptor seems to influence the dynamics within the protein complex, in particular the relative movements of the two *α*-helices, G*α*_1_ and G*α*_2_.

## 1. Introduction

The interaction between major histocompatibility complexes (MHCs) and T-cell receptors (TCRs) plays a key role in triggering adaptive immune responses. TCRs bind the highly polymorphic MHC proteins that present peptide fragments (p) derived from the host proteome, pathogens, or tumour antigens on the cell surface. TCR and pMHC represent the core of the immunological synapse that in turn comprises many proteins, both membrane-bound and in the cytosol that could relay signals and/or act as an adjustable screw to fine-tune TCR sensitivity. Cluster of differentiation 8 (CD8) is a transmembrane, mostly heterodimeric glycoprotein that functions as a coreceptor for the TCR. It is mainly expressed by cytotoxic T-cells (T_C_), but is also found on natural killer cells, cortical thymocytes, and dendritic cells [1]. The extracellular domain of CD8 binds to the *α*_3_-domain of the MHC class I heavy chain [2]. It is well-known that CD8 and CD4 coreceptors are able to enhance T-cell responses to antigen stimulation [3–5]. Also, when subjected to an immune response, CD8^+^ T-cells can substantially increase in sensitivity by the mechanism of functional avidity maturation, that is, maturation of strength of multivalent antigen-antibody binding [6–8].

According to the literature, the major mechanism for stabilizing TCR-pMHC interaction by CD8 is the CD8-MHC interaction that increases the TCR-pMHC rebinding probability [9, 10]. A less obvious mechanism is stated by Borger et al. [11] and includes affected binding rates of TCR-pMHC. They propose a two-stage reversible reaction mechanism of pMHC with either TCR or CD8, similar to the mechanism found by Liu et al. [12].

**Figure 1:**
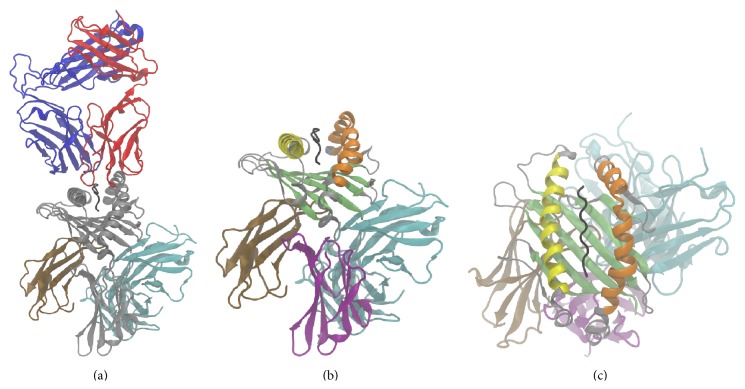
Structure description. (a) Cartoon representation of the TCR/pMHC/CD8 system: MHC (grey), *β*_2_-microglobulin (ochre), peptide (black), TCR *α*-chain (red), TCR *β*-chain (blue), and CD8 *α*_1_ and CD8 *α*_2_ (cyan). (b, c) Cartoon representation of the pMHC/CD8 complex: *α*-helix G*α*_1_ (yellow), *α*-helix G*α*_2_ (orange), *α*_3_-domain (purple), *β*-sheet (lime), and CD8 *α*_1_ and CD8 *α*_2_ (cyan).

Molecular dynamics (MD) simulations of TCR/pMHC (PDB ID: 3KPS) and TCR/pMHC (3KPS) plus CD8*αα* homodimer were performed. The topology of the TCR/pMHC complex with and without a CD8 coreceptor is shown in Table 1 and Figure 1. We chose to monitor the relative movements of MHC *α*-helices, G*α*_1_ and G*α*_2_, with and without the presence of CD8 (as these helices constitute part of the binding cleft for the peptide) and of the MHC *α*_3_-domain relative to the whole CD8 (since the *α*_3_-domain is the binding site for the CD8-coreceptor).

## 2. Methods

### 2.1. Molecular Modelling

The structure of LC13 TCR, ABCD3 peptide, and MHC (TCR/pMHC) of HLA-B^*∗*^44:05 type has been resolved (PDB-ID: 3KPS, www.pdb.org). Also, molecular structures of CD8 coreceptors bound to MHCs are available (PDB-ID: 1AKJ).

To our knowledge, a TCR/pMHC/CD8 complex has not yet been cocrystallized. To model the TCR/pMHC/CD8 complex we localized the CD8/MHC binding site in the 1AKJ crystal structure by finding all *C*_*α*_ atoms of the MHC within the range of 0.8 nm to CD8. Structures of TCR/pMHC (3KPS) and MHC/CD8 (1AKJ) were merged into one file, both MHC binding sites superimposed so as to minimize RMSD in a least-squares sense and the MHC molecule from the MHC/CD8 complex deleted to get the TCR/pMHC/CD8 complex; see Figure 1.

### 2.2. Molecular Dynamics Simulations

Molecular dynamics simulations were performed with GROMACS 4.0.7 [13] using the gromos53a6 force field. The whole system counts about 274000 atoms, including the solvent (protein atoms only: about 8500), within a simulation box sized 13 × 13.5 × 16.5 nm^3^, to ensure 2 nm minimal distance between the protein atoms and the box walls, and with periodic boundary conditions imposed. The solvent was described with the SPC water model [14], the system neutralized at a salt concentration of 0.15 mol/L, and its energy was minimized by the steepest-descent method. The temperature was then gradually increased to 310 K within a 100 ps position-restraint simulation. Temperature was controlled by a Berendsen-thermostat with a time constant of 0.1 ps and the pressure controlled by a Berendsen-barostat set to 1 bar with a time constant of 0.5 ps, both chosen for being the most efficient in the beginning of the simulation. Constraints on all bonds were imposed with the LINCS algorithm [15], and the particle mesh Ewald (PME) method [16] was used to compute the long-range electrostatic interactions, with van der Waals and Coulomb cutoff radii of 1.4 nm. For the MD simulation runs of 200 ns with a time step of 5 fs, enabled by using virtual sites for hydrogen atoms, the thermostat was set to v-rescale, with the same time constants in order to guarantee the generation of a proper canonical ensemble [13]. Coordinates were written every 50 ps, giving thus rise to 4000 frames. Prior to the analysis of domain movements, translational and rotational motions relative to the energy-minimized protein structure were removed.

### 2.3. Relative Location of Domains

Within the biomolecules we consider the* C*_*α*_ atoms in the backbones of protein chains. These* C*_*α*_ atoms are addressed via their indices to define domains or even subdomains; see Table 1. We define a first domain, *V*, by enumerating the* C*_*α*_ atoms contained; for example, to select the *α*-helix G*α*_1_ we have *V* = {59,60,…, 83}. Similarly, we define a second domain, *W*. Note that domains may consist of several parts such as the *β*-sheet; see Table 1.

Considering a domain *V* containing *N*_*V*_ *C*_*α*_ atoms with Cartesian coordinates **x**_*i*_(*f*) in MD-frame *f*, the coordinates of its geometrical center are given by

(1) $$\begin{array}{c} {\overset{-}{x}}_{V}\left(\;f\;\right)=\frac{1}{N_{V}}\underset{i\in V}{\sum }x_{i}\left(\;f\;\right). \end{array}$$

The distance between the centers of two domains, *V* and *W*, in MD-frame *f* is then

(2) $$\begin{array}{c} d\left(\;f\;\right)=\left\|\;{\overset{-}{x}}_{W}\left(\;f\;\right)-{\overset{-}{x}}_{V}\left(\;f\;\right)\;\right\|. \end{array}$$

Both domains are shifted with their mean* C*_*α*_ coordinates into the origin before defining their relative orientation.

#### 2.3.1. Rigid Axes within Deformable Domains

To quantify the relative orientation of two domains one has to bear in mind that each domain is deformed from time step to time step of an MD simulation and hence no unique frame of reference can easily be assigned as is possible for a rigid body. Often a principal component analysis (PCA) is performed to obtain three main characteristic axes of a given domain [17–19]. However, principal components are not fully rigorously defined: for example, the orientation of the first PC-eigenvector may swap by nearly 180° for virtually the identical coordinates, just because of numerical noise. Similarly, the second and third PC-eigenvectors may interchange roles from time to time for an atomic domain almost cylindrical in shape.

**Figure 2:**
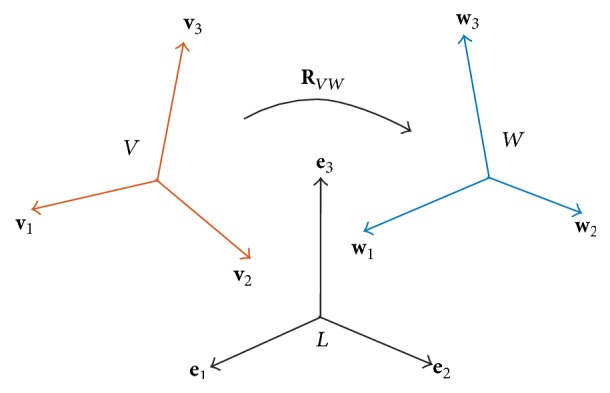
Relative orientation of two submolecular domains. Orthonormal eigenvectors for domains* V* and *W* and standard basis for the laboratory system* L*. Rotation matrix **R**_*VW*_ transforms eigenvectors from domain *V* into domain *W*. Note that eigenvectors share the same coordinate system origin. For better visualization, eigenvectors are displayed as if they had different origins.

In order to avoid such artifacts we refrained from computing principal components repeatedly for each MD time step and adopted the following procedure, see Figure 2:

(1) For domain *V* we select a reference frame, *k*_*V*_, from the whole trajectory. This is done by computing the sum of RMSD-displacements of *V* relative to itself [20] over all frames of the trajectory and adopting the frame *k*_*V*_ with minimum sum of RMSD. This frame is in a geometrical sense considered most “central” for domain *V* within the whole trajectory.
(2) The* C*_*α*_ coordinates of domain *V* at frame *k*_*V*_ are subjected to a PCA, yielding the orthogonal matrix **T**_*V*_(*k*_*V*_) = [**v**_1_ **v**_2_ **v**_3_]_*k*_*V*__ of three orthonormal eigenvectors **v**_1_, **v**_2_, **v**_3_ of the covariance matrix of the coordinates of* C*_*α*_ atoms in domain *V* at frame *k*_*V*_. These vectors define a reference frame (i.e., a local coordinate system) for domain *V*.
(3) Steps (1) and (2) are performed also for domain *W*, yielding the respective central frame *k*_*W*_ with an eigenvector matrix **T**_*W*_(*k*_*W*_) = [**w**_1_ **w**_2_ **w**_3_]_*k*_*W*__ and a local coordinate system for *W*.

#### 2.3.2. Computing Robust Relative Orientations

Given all MD-frames *f* of a trajectory, the relative orientation of domains *V* and *W* is computed as follows:

(a) For each frame *f* we compute the transformation of all coordinates **x**_*V*_(*k*_*V*_) of domain *V* from its position within the central frame into its position at frame *f* according to minimum RMSD using Kabsch's method [20].
(b) The rotational part **R**_*V*_(*f*) of the above transformation is applied to the eigenvectors of domain *V* at frame *k*_*V*_ (the reference frame) to obtain the position of the eigenvectors of *V* at frame *f*: **T**_*V*_(*f*) = [**v**_1_ **v**_2_ **v**_3_]_*f*_.
(c) Steps (a) and (b) are performed also for domain *W*, yielding **R**_*W*_(*f*) and transformed eigenvectors **T**_*W*_(*f*) = [**w**_1_ **w**_2_ **w**_3_]_*f*_ for each frame *f*.
(d) For each frame *f* we note that the directional relation between the two sets of eigenvectors [**v**_1_ **v**_2_ **v**_3_]_*f*_ and [**w**_1_ **w**_2_ **w**_3_]_*f*_ can be represented via a rotation matrix **R**_*VW*_(*f*) as
  (3) $$\begin{array}{c} T_{W}\left(\;f\;\right)=R_{VW}\left(\;f\;\right)\cdot T_{V}\left(\;f\;\right) \end{array}$$
 which also characterizes the relative orientation of both domains. From (3) we obtain
  (4) $$\begin{array}{c} R_{VW}\left(\;f\;\right)=T_{W}\left(\;f\;\right)\cdot T_{V}^{-1}\left(\;f\;\right)=T_{W}\left(\;f\;\right)\cdot T_{V}^{T}\left(\;f\;\right) \end{array}$$
 since the inverse of an orthogonal matrix equals its transpose. Rewriting the matrix **R**_*VW*_(*f*) in terms of its column vectors
  (5) $$\begin{array}{c} R_{VW}\left(\;f\;\right)=\left[\;\left(\begin{array}{c} r_{1x}\\ r_{1y}\\ r_{1z} \end{array}\right)\left(\begin{array}{c} r_{2x}\\ r_{2y}\\ r_{2z} \end{array}\right)\left(\begin{array}{c} r_{3x}\\ r_{3y}\\ r_{3z} \end{array}\right)\;\right] \end{array}$$
 the Euler angles in *x*-convention [21] between the two sets of the orthonormal eigenvectors [**v**_1_ **v**_2_ **v**_3_]_*f*_ and [**w**_1_ **w**_2_ **w**_3_]_*f*_ can be read as
  (6) α=arccos⁡r3y1−r3z2,β=arccos⁡r3z,γ=arccos⁡r2y1−r3z2
 with all quantities depending on frame* f* (dependencies suppressed in the notation).

As a result, *d*(*f*), *α*(*f*), *β*(*f*), and *γ*(*f*) define the relative spatial relation between domains *V* and *W* for MD frame *f*.

## 3. Results

### 3.1. Relative Movements CD8-MHC

**Figure 3:**
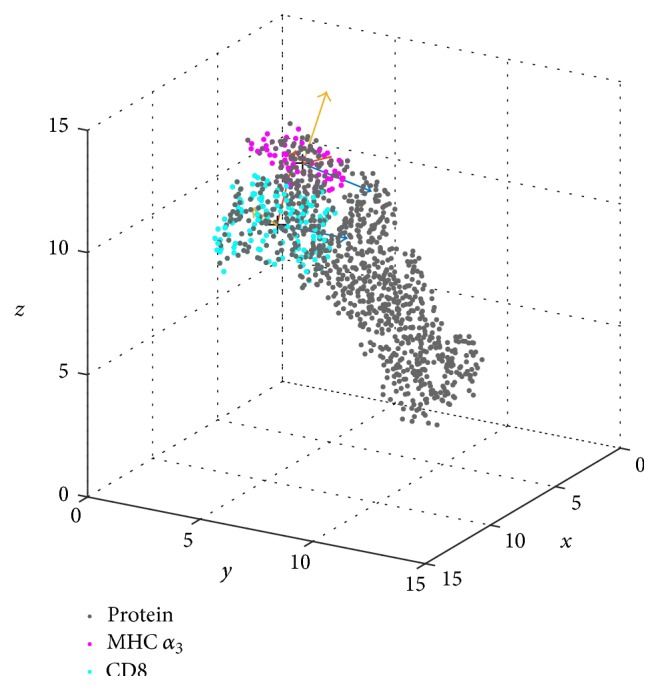
Domains CD8 and MHC *α*_3_ with local eigenvectors. Domain CD8 is shown in cyan, *α*_3_ in purple, and the remaining parts of the MHC as well as the TCR (labelled “protein”) in black. Eigenvectors (**v**_1_, **v**_2_, **v**_3_ and **w**_1_, **w**_2_, **w**_3_, resp.) are shown for the first frame of the trajectory and colored (**v**_1_, **w**_1_: blue, **v**_2_, **w**_2_: red, **v**_3_, **w**_3_: yellow) for each of the domains.

As a first pair of domains we considered the CD8 homodimer (CD8 *α*_1_, CD8 *α*_2_) as domain *V* and domain *α*_3_ of the MHC (see Table 1) as domain *W*. Domain *α*_3_ is the binding site for CD8 (see Figure 3) and therefore most interesting regarding relative movements.

#### 3.1.1. Relative Distance

**Figure 4:**
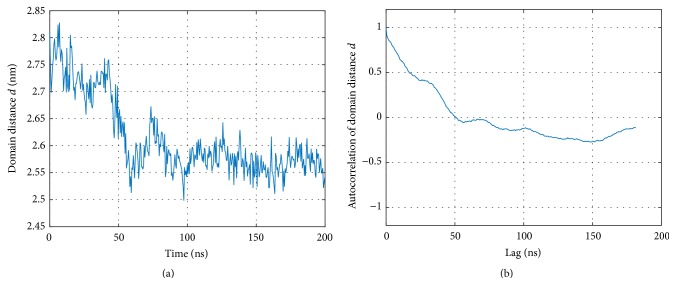
Distance between CD8 and MHC *α*_3_. (a) Distances computed according to (2). (b) Autocorrelation of distance values.

The distance *d* between domains, (2), was computed over the time; see Figure 4. This distance, initially around 2.75 nm (as modelled, based on the crystallographic structure), gradually decreases to about 2.55 nm during the first 50 ns of the simulation. Apparently, dynamics lets CD8 get somewhat closer to the TCR/pMHC complex.

#### 3.1.2. Relative Orientation

**Figure 5:**
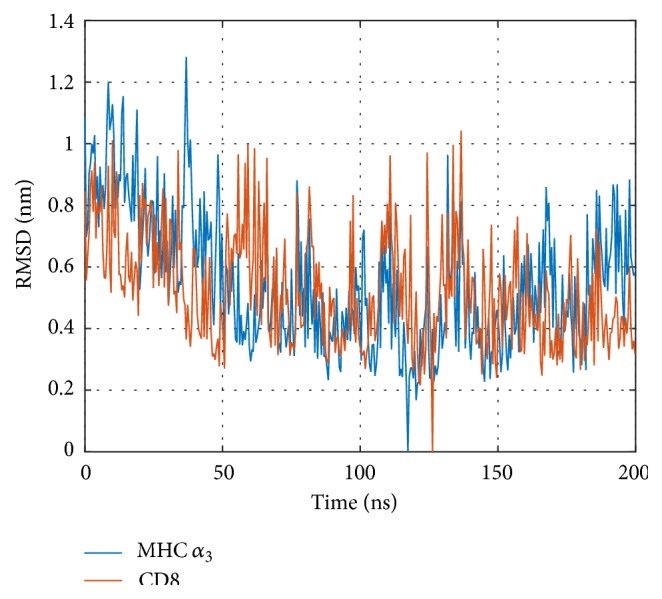
RMSD from central frame of each domain to all other frames of trajectory. Note that RMSD is zero for the respective reference frames against themselves by definition (*k*_*V*_ = 237 ≡ 117.30 ns and *k*_*W*_ = 255 ≡ 126.25 ns).

For each domain, the sum of RMSD of each frame to all other frames of the trajectory was computed to determine the central (reference) frames, *k*_*V*_ and *k*_*W*_; see Figure 5.

Relative positions of both domains were then computed for each frame (4000 all in all) as outlined above leading to the following results; see Section 2.3.2.

**Figure 6:**
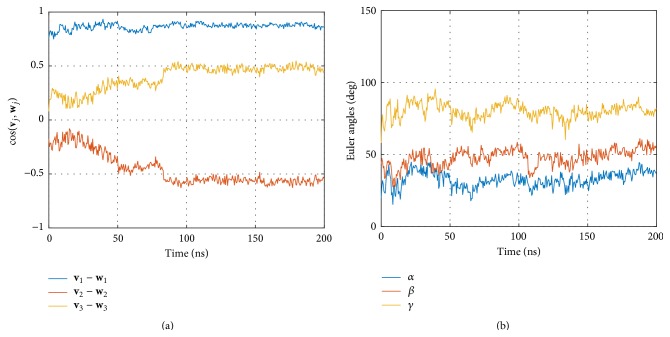
Relative Orientation of CD8 and MHC *α*_3_. (a) Cosine values between corresponding eigenvectors (see legend box) of both domains over time. Every 10th frame is plotted. (b) Euler angles for the same data.

A first and direct measure is provided by the orientation cosines between corresponding eigenvectors; see Figure 6(a). They clearly reflect an initial phase different from the remaining trajectory, corresponding to the phase of decreasing interdomain distance, already seen in Figure 4. Interestingly, this effect is not at all revealed by the Euler angles; see Figure 6(b).

**Figure 7:**
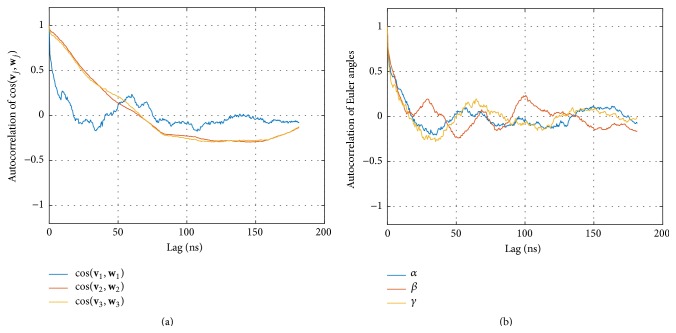
Autocorrelation of relative directions of CD8 and MHC *α*_3_. (a) Autocorrelation of cosine values between corresponding eigenvectors (see legend box) of both domains over time. Every 10th frame is plotted. (b) Autocorrelation of Euler angles for the same data.

To further characterize the evolution of the geometry of the complex with time we computed the autocorrelation functions (ACFs) for cosines between eigenvectors and for Euler angles; see Figure 7(b). Euler angles exhibited relatively short autocorrelations, passing through zero already at approximately 20 ns, and oscillating around zero for larger time lags.

A completely different picture results from inspecting the ACFs of the cosines between corresponding eigenvectors; see Figure 7(a). While the relative orientation of the eigenvectors corresponding to the largest eigenvalues, **v**_1_ and **w**_1_, has only a short memory (the ACF passes through zero around 20 ns), a massively prolonged memory is seen for both smaller eigenvectors **v**_2_ versus **w**_2_ and **v**_3_ versus **w**_3_: they exhibit a long negative tail and do not become stochastic throughout the whole simulation time. This reflects the fact already seen in the time course itself (Figure 6(a)): a long equilibration phase extends up to about 100 ns (which amounts to half the simulation time).

### 3.2. Relative Movements of Two MHC *α*-Helices

**Figure 8:**
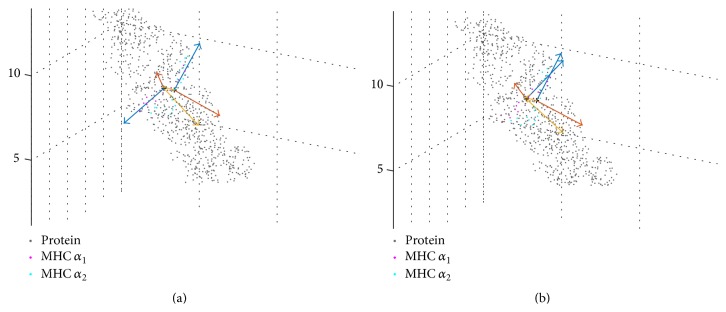
Eigenvectors switching orientations between frames. (a) Eigenvectors of first frames. (b) Eigenvectors of central frames *k*_*V*_ and *k*_*W*_. If calculated from the atomic coordinates, the first eigenvectors (**v**_1_,** w**_1_: blue) result with almost opposite orientations in the first frame but very similar orientations in the central frames of each domain. Note that eigenvectors refer to different frames *k*_*V*_ and *k*_*W*_, respectively, but atoms of the molecule are plotted only once. Coloring:** v**_1_,** w**_1_: blue;** v**_2_,** w**_2_: red, and** v**_3_,** w**_3_: yellow.

The two *α*-helices of the MHC, G*α*_1_ and G*α*_2_, together with a *β*-floor form the binding cleft for the peptide. To evaluate their relative motion we chose the helices as domains *V* and *W*, located the central (reference) frames (*k*_*V*_ = 279 ≡ 138.4 ns and *k*_*W*_ = 345 ≡ 171.2 ns) of each, and computed corresponding eigenvectors; see Figure 8(b). It is interesting to see that the first eigenvectors **v**_1_(*k*_*V*_) and **w**_1_(*k*_*V*_) have similar directions and orientations, when computed for the central frames. As opposed to this, they show almost opposite directions when computed from the first frame of the trajectory; see Figure 8(a). These opposite directions of eigenvectors are formally obtained from the very same matrix algebra, although the domains themselves have by no means turned upside down, as one can see by simple inspection. We have addressed this issue in Section 2 and display an example here. Our method of fitting reference domains via the Kabsch method has been designed to avoid these effects. In fact, the eigenvectors **v**_1_(1) and **w**_1_(1) we actually use for frame 1 (and for all other frames) have orientations similar to **v**_1_(*k*_*V*_) and **w**_1_(*k*_*V*_), except for the actual, relative movements of both domains.

**Figure 9:**
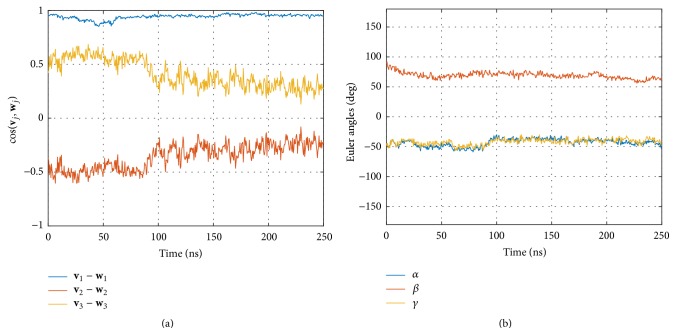
Relative movements of helices G*α*_1_ and G*α*_2_ in absence of CD8. (a) Cosines between corresponding eigenvectors. (b) Euler angles between corresponding eigenvectors.

Relative motions of G*α*_1_ and G*α*_2_ are characterized by cosines (see Figure 9(a)) and Euler angles (Figure 9(b)) between eigenvectors attached to each domain. Cosines as well as Euler angles reveal a correlation in movements of eigenvectors 2 and 3. These eigenvectors point away from the axis of the helices at right angles, and their correlated changes indicate a synchronized oscillating “rolling” of both helices. In fact this kind of movement is also evident when visually inspecting the trajectories in VMD [22].

**Figure 10:**
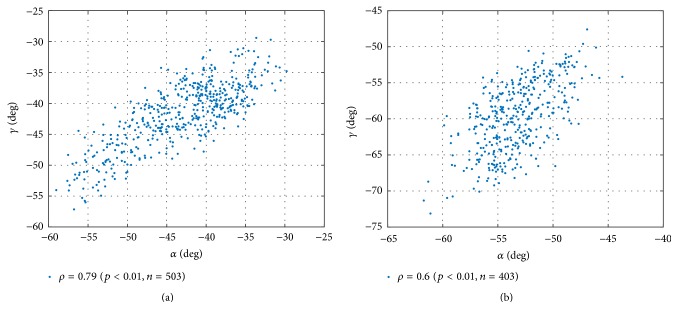
The presence of CD8 changes the relative movement of domains. (a) Scatter plot of Euler angles *α* and *γ* between domains MHC *α*_1_ and *α*_2_ in the absence of CD8. (b) Scatter plot of the Euler angles *α* and *γ* with CD8 present.

The above finding is nicely quantified via the correlation coefficient *ρ* = 0.79 (with *p* < 0.01 and *N*_frames_ = 503); see Figure 10(a).

### 3.3. Impact of CD8 Presence

We have also analyzed the relative motions of G*α*_1_ and G*α*_2_ from our MD simulation of TCR/pMHC/CD8 with CD8 attached to the MHC and compared it with the above results without CD8. Interestingly, the relative motions do not differ significantly (figures not shown); however, the correlation of “rolling” oscillations is almost lost in the presence of CD8: correlation coefficient *ρ* = 0.6 (with *p* < 0.01 and *N*_frames_ = 406); see Figure 10(b). To check if this difference in correlation coefficients is statistically significant, we computed the 95%-confidence intervals [23], resulting in [0.755,0.821] for *ρ* = 0.79 (*N*_frames_ = 503) and [0.553,0.659] for *ρ* = 0.6 (*N*_frames_ = 406). These intervals do not overlap and the difference in correlation coefficients may thus be considered statistically significant.

## 4. Discussion

The methodological parts of this work describe a new computational technique to obtain relative orientations of intramolecular domains. In the application parts this method is used to analyze the molecular dynamics of two systems, TCR/pMHC and TDC/pMHC/CD8, respectively.

### 4.1. Methods to Characterize Relative Orientations

There is a plethora of ways to characterize relative movements of intramolecular domains (e.g., [17–19, 24–27]). The most direct one is to compute average distances between groups of atoms, as they change over time. We did this by computing the distance between CD8 and MHC *α*_3_; see Figure 4. By appropriate selection of target groups, some basic information can be obtained also on relative orientations.

In this work we present a more sophisticated approach by attaching local coordinate systems (of eigenvectors) to each domain and calculating the rotational relations between them. It is a well-known drawback of eigenvector-based techniques that the eigenvector* orientation* is not well defined and may suddenly switch into almost opposite directions. Up to now, this was in most cases mended by some logical condition in the code selecting the appropriate orientation with reference to some atoms that have to be individually specified. These drawbacks are even more severe when eigenvectors are computed from internally deformable sets of data points, such as MD-frames.

We have solved this problem by computing eigenvectors only once (for each domain) from a very specific frame (the central frame), see Section 2.3.1. In this first step, the orientation of both systems of eigenvectors may be corrected if desired. Thereafter, relations will remain stable without any intervention on the side of the researcher. Stability of local coordinate systems is achieved by fitting the atoms within each domain and carrying along the eigenvectors accordingly. This results in robust relative orientations.

Note that although the method seems computationally demanding, it is in fact fast. The reason is that the Kabsch algorithm is a direct matrix operation, not an iterative procedure, as one might expect. It requires no larger computational effort than singular value decomposition.

Once mutual orientations have been computed (rotation matrix), some attention was given to select suitable quantities to characterize relative orientations. We presented cosines between corresponding eigenvectors, since they can easily be interpreted. In addition, we used Euler angles as a well-known concept for relative orientations of rigid bodies.

### 4.2. Unifying Orientations

PCA as such yields eigenvectors unique in directions but ambiguous in orientation. For example, the first eigenvector **v**_1_ may also result as **v**_1_^*∗*^ = −**v**_1_, merely as a consequence of minute numerical noise in data substantially equal. The same is true for **v**_2_. The third eigenvector always has to satisfy

(7) $$\begin{array}{c} v_{3}=v_{1}\times v_{2} \end{array}$$

and is hence determined by **v**_1_ and **v**_2_. When comparing the relative orientation of two domains via their eigenvectors, orientation ambiguity has to be coped with, which could be done as follows:

(i) After performing a PCA for the reference frame *k*_*V*_ of domain *V*, results are manually inspected and **v**_1_ given a well-defined orientation within domain *V*. This can be accomplished by selecting two* C*_*α*_ atoms and setting the orientation of **v**_1_ such that a positive cosine of the angle between **v**_1_ and the vector joining the two* C*_*α*_ atoms is obtained.
(ii) The same is done for **v**_2_, with a second pair of appropriate* C*_*α*_ atoms selected. **v**_3_ is computed via the cross product of **v**_1_ and **v**_2_; see above.

In order to arrive at standardized eigenvectors allowing for comparison between different MD-runs the resulting eigenvectors should be reoriented (if necessary) after performing PCA for the reference frame *k*_*W*_ of domain *W*: a criterion for reorientation could be positive cosines with the eigenvectors of domain *V*:

(8) v1·w1≥0,v2·w2≥0.

Note that flipping the orientation of eigenvectors between different frames of a trajectory is avoided intrinsically by our procedure (fitting of domains rather than repeatedly performing PCA to successive frames). However, initially selecting appropriate and definite orientations is only guaranteed by the above precautions.

### 4.3. Application of the New Methods to CD8 Coreceptor Dynamics

Inspecting the distance between CD8 and MHC *α*_3_ over time (Figure 4(a)) we clearly observe an equilibration phase pertaining up to 50 ns, during which distance decreases. This observation suggests that more extensive MD simulations than those presented in this work are necessary to reliably investigate equilibrium properties of this system. This finding is also supported by the autocorrelation function, which passes through zero around 60 ns and shows a long negative tail afterwards; see Figure 4(b).

The existence of an equilibration phase raises the importance of a comparison of the two presentation methods employed: cosines between eigenvectors and Euler angles (see Figure 6). Clearly, cosines reflect the fact that there is a relaxation phase, while the Euler angles do not. Interestingly, the eigenvectors corresponding to the main extensions (**v**_1_, **w**_1_) fail to indicate the relaxation but (**v**_2_, **w**_2_) and (**v**_3_, **w**_3_) clearly do. This indicates that the relaxation is made up of some rotation around the major axes, **v**_1_ and **w**_1_, respectively.

This surprising finding is supported by comparing the corresponding autocorrelation functions; see Figure 7. Euler angles (Figure 7(b)) lose memory already after 20 ns and the ACF then oscillates around zero. As opposed to this, cosines between eigenvectors corresponding to the smaller components ((**v**_2_, **w**_2_) and (**v**_3_, **w**_3_)) show excessively long autocorrelations, pertaining throughout the whole simulation time. This finding, even more than the 50 ns equilibration of distance, indicates the necessity of additional MD simulations to arrive at a more adequate sampling.

As a most interesting result, we found that the presence of CD8 seems to influence the dynamics within the MHC, in particular the relative movements of the two *α*-helices, G*α*_1_ and G*α*_2_. In this case, Euler angles proved the more sensitive tool. Negative correlation between cosines of (**v**_2_, **w**_2_) and (**v**_3_, **w**_3_) was in both cases beyond *ρ* < −0.9, and presence or absence of CD8 did not make much difference. For the Euler angles, however, we obtained the nice reduction of movement-correlation induced by the presence of CD8; see Figure 10.

All in all, both sets of orientation parameters presented (cosines and Euler angles) seem to have their merits and weaknesses that have to be explored in many more situations to arrive at a comprehensive judgment.

## Figures and Tables

**(a):** 

Chain	Type	Length in *C*_*α*_	*C*_*α*_ index
Chain A	MHC	276	1–276
Chain B	*β*_2_-microglobulin	99	277–375
Chain C	Peptide	9	376–384
Chain D	TCR, *α*-chain	201	385–585
Chain E	TCR, *β*-chain	241	586–826
Chain F	CD8 *α*_1_	114	827–940
Chain G	CD8 *α*_2_	114	941–1054

**(b):** 

Secondary structures	Chain	Length in *C*_*α*_	*C*_*α*_ index
*α*-helix G*α*_1_	A	25	59–83
*α*-helix G*α*_2_	A	31	141–170
*α*_3_-domain	A	92	184–275
*β*-sheet	A	52	2–13, 21–29, 30–37, 93–103, 110–118, 124–127
TCR *α*_var_	D	104	385–488
TCR *β*_var_	E	117	586–702

Structural elements are given in terms of consecutive numbers of *C*_*α*_ atoms, renumbered throughout the whole modelled TCR/pMHC/CD8 complex, as if the complex as a whole was taken from one single PDB file.

Molecule 1 (TCR/pMHC): Chain A–Chain E. Molecule 2 (TCR/pMHC/CD8): Chain A–Chain G.
